# The *Tara* Pacific expedition—A pan-ecosystemic approach of the “-omics” complexity of coral reef holobionts across the Pacific Ocean

**DOI:** 10.1371/journal.pbio.3000483

**Published:** 2019-09-23

**Authors:** Serge Planes, Denis Allemand, Sylvain Agostini, Bernard Banaigs, Emilie Boissin, Emmanuel Boss, Guillaume Bourdin, Chris Bowler, Eric Douville, J. Michel Flores, Didier Forcioli, Paola Furla, Pierre E. Galand, Jean-François Ghiglione, Eric Gilson, Fabien Lombard, Clémentine Moulin, Stephane Pesant, Julie Poulain, Stéphanie Reynaud, Sarah Romac, Matthew B. Sullivan, Shinichi Sunagawa, Olivier P. Thomas, Romain Troublé, Colomban de Vargas, Rebecca Vega Thurber, Christian R. Voolstra, Patrick Wincker, Didier Zoccola, the Tara Pacific Consortium

**Affiliations:** 1 Laboratoire d’Excellence “CORAIL,” PSL Research University: EPHE-UPVD-CNRS, USR 3278 CRIOBE, Université de Perpignan, Perpignan Cedex, France; 2 Research Federation for the study of Global Ocean Systems Ecology and Evolution, FR2022/*Tara* Oceans-GOSEE, Paris, France; 3 Centre Scientifique de Monaco, Monte Carlo, Principality of Monaco; 4 Shimoda Marine Research Center, Shimoda, Japan; 5 School of Marine Sciences, University of Maine, Orono, Maine, United States of America; 6 Sorbonne Université, Institut de la Mer de Villefranche sur mer, Laboratoire d'Océanographie de Villefranche, Villefranche-sur-Mer, France; 7 Institut de Biologie de l'Ecole Normale Supérieure (IBENS), Ecole normale supérieure, CNRS, INSERM, Université PSL, Paris, France; 8 Laboratoire des Sciences du Climat et de l’Environnement, LSCE/IPSL, CEA-CNRS-UVSQ, Université Paris-Saclay, Gif-sur-Yvette, France; 9 Weizmann Institute of Science, Dept. Earth and Planetary Science, Rehovot, Israel; 10 Université Côte d'Azur-CNRS-INSERM, IRCAN, Medical School, Nice, France and Department of Medical Genetics, CHU of Nice, Nice, France; 11 Sorbonne Université, CNRS, Laboratoire d’Ecogéochimie des Environnements Benthiques (LECOB), Observatoire Océanologique de Banyuls, Banyuls sur mer, France; 12 Sorbonne Université Laboratoire d’Océanographie Microbienne LOMIC, UMR 7621, Observatoire Océanologique de Banyuls, Banyuls sur mer, France; 13 La Fondation *Tara* Expéditions, “Base *Tara*” 11, Paris, France; 14 PANGEA, Data Publisher for Earth and Environment Science, Bremen, Germany; 15 MARUM—Center for Marine Environmental Sciences, Universität Bremen, Bremen, Germany; 16 Génomique Métabolique, Genoscope, Institut François Jacob, CEA, CNRS, Université Evry, Université Paris-Saclay, Evry, France; 17 Sorbonne Université, CNRS, Station Biologique de Roscoff, AD2M, UMR 7144, ECOMAP, Roscoff, France; 18 Departments of Microbiology and Civil, Environmental and Geodetic Engineering, The Ohio State University, Columbus, Ohio, United States of America; 19 Department of Biology and Swiss Institute of Bioinformatics, ETH Zürich, Zürich, Switzerland; 20 Marine Biodiscovery Laboratory, School of Chemistry and Ryan Institute, National University of Ireland, Galway (NUI Galway), Galway, Ireland; 21 Department of Microbiology, Oregon State University, Corvallis, Oregon, United States of America; 22 Department of Biology, University of Konstanz, Konstanz, Germany

## Abstract

Coral reefs are the most diverse habitats in the marine realm. Their productivity, structural complexity, and biodiversity critically depend on ecosystem services provided by corals that are threatened because of climate change effects—in particular, ocean warming and acidification. The coral holobiont is composed of the coral animal host, endosymbiotic dinoflagellates, associated viruses, bacteria, and other microeukaryotes. In particular, the mandatory photosymbiosis with microalgae of the family Symbiodiniaceae and its consequences on the evolution, physiology, and stress resilience of the coral holobiont have yet to be fully elucidated. The functioning of the holobiont as a whole is largely unknown, although bacteria and viruses are presumed to play roles in metabolic interactions, immunity, and stress tolerance. In the context of climate change and anthropogenic threats on coral reef ecosystems, the *Tara* Pacific project aims to provide a baseline of the “-omics” complexity of the coral holobiont and its ecosystem across the Pacific Ocean and for various oceanographically distinct defined areas. Inspired by the previous *Tara* Oceans expeditions, the *Tara* Pacific expedition (2016–2018) has applied a pan-ecosystemic approach on coral reefs throughout the Pacific Ocean, drawing an east–west transect from Panama to Papua New Guinea and a south–north transect from Australia to Japan, sampling corals throughout 32 island systems with local replicates. *Tara* Pacific has developed and applied state-of-the-art technologies in very-high-throughput genetic sequencing and molecular analysis to reveal the entire microbial and chemical diversity as well as functional traits associated with coral holobionts, together with various measures on environmental forcing. This ambitious project aims at revealing a massive amount of novel biodiversity, shedding light on the complex links between genomes, transcriptomes, metabolomes, organisms, and ecosystem functions in coral reefs and providing a reference of the biological state of modern coral reefs in the Anthropocene.

## Abstract 2

This Community Page article presents the Tara Pacific expedition, which aims to shed light on the complex links between genomes, transcriptomes, metabolomes, organisms, and ecosystem functions in coral reefs, and providing a reference of the biological state of modern reef systems.

## Introduction

The 20th century has seen the earth enter into the now widely called Anthropocene [1]. Anthropogenically induced changes are happening on both global and local scales and are altering the physiology of organisms and ecosystems by modifying the entire earth’s physical, chemical, and biological processes [2]. Among marine ecosystems, coral reefs have the unfortunate privilege of being highly sensitive to these environmental modifications [3]. In particular, the thermally mediated process of coral bleaching—i.e., the loss of the obligate photosynthetic microalgal endosymbionts—is increasingly decimating corals. Projections estimate that approximately 25% of reefs have already been lost, and up to 99% will be threatened and dramatically transformed by 2050 [4]. Even though they cover only approximately 0.2% of the ocean’s surface [5], coral reefs harbor approximately 30% of ocean biodiversity [6], providing ecological services (fisheries, tourism, coastal protection) to nearly 1 billion people [7], and are estimated to be worth USD 30 billion per year [8].

The late 20th century has also seen our world entering into the “-omics” revolution sparked by the development of high-throughput analyses of DNA, RNA, proteins, and metabolites. This revolution has changed our approach to investigate organisms, and it is about to change the descriptions of ecosystems as genes-to-ecosystem modeling improves [9]. It will advance our capabilities to investigate the biodiversity and functioning of our oceans in a holistic way. Holistic approaches, interrogating components across the various levels of organization of an ecosystem, appear accessible today, as exemplified in the *Tara* Oceans project [10], which continues to decipher biodiversity and structural networks across all plankton organisms in the ocean [11].

*Tara* Pacific is a unique scientific expedition inspired by earlier maritime explorations that uncovered the unchartered territories of marine biodiversity. Coral reef research started with the contribution of Charles Darwin during “the voyage of the *Beagle*” (1831–1836), when Darwin explored many reefs in the Indian and Pacific Oceans and established his biophysical theory of the formation of coral reefs and atolls [12]. He also highlighted the paradoxical high diversity of reef organisms living in a nutrient-poor system. Later, the Great Barrier Reef Expedition (1928–1929) led by Charles Yonge was a landmark in coral reef research, as it established the scientific basis of coral physiology (nutrition, symbiosis, growth, etc.). The “polypifers,” later called coral polyps, were then demonstrated to host a large population of organisms (protists, viruses, bacteria, archaea, and all sorts of unicellular and multicellular eukaryotes), which are intimately involved in the physiology of the coral animal host in a complex and still mysterious system named the holobiont [13].

**Fig 1:**
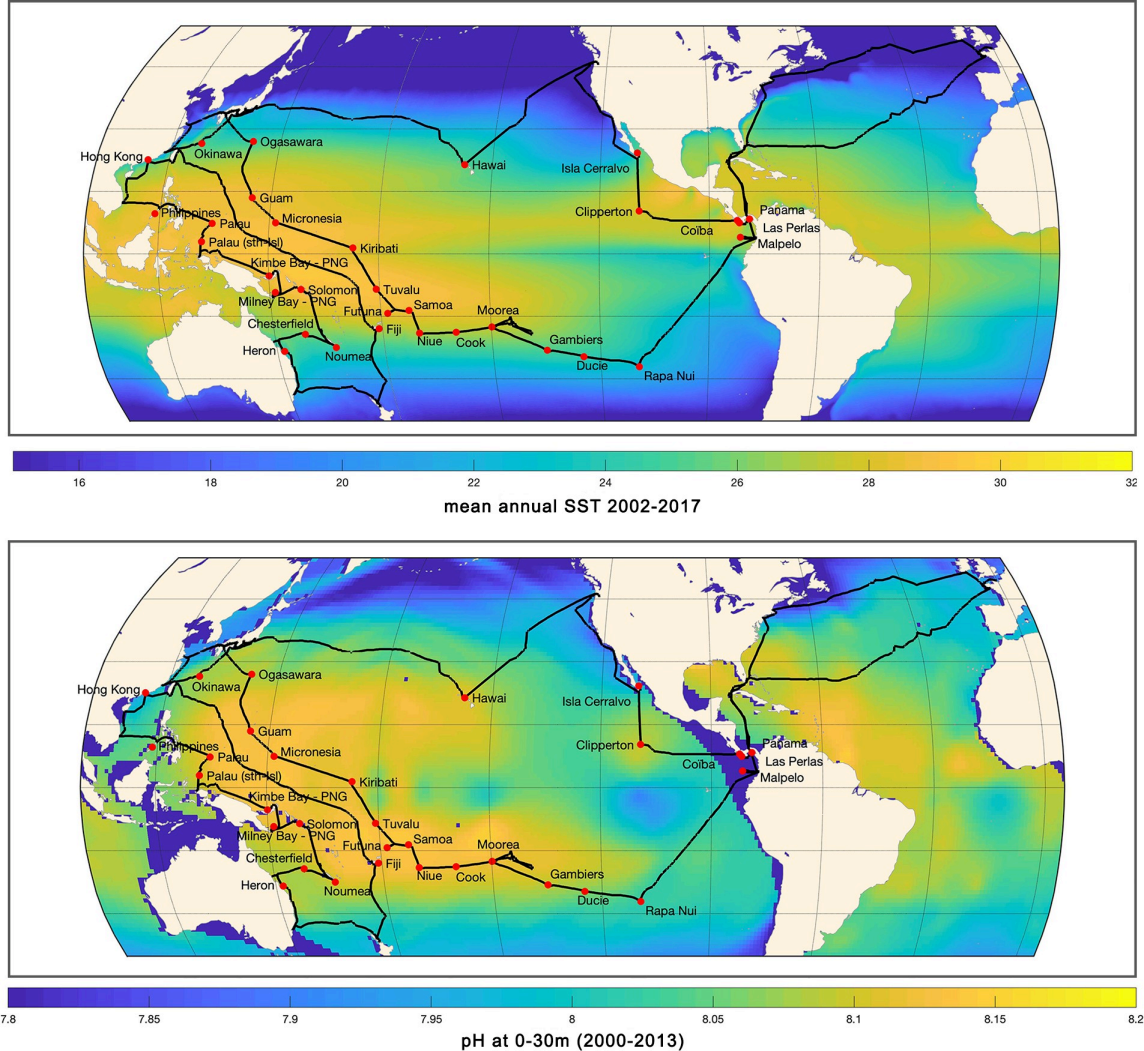
Map showing the route of the *Tara* Pacific expedition and the sampling sites (red spots) throughout the Pacific Ocean (July 2016 to October 2018) as well as the mean annual SST (top) and pH (bottom). Global annual SSTs were extracted from the MODIS-Aqua satellite and correspond to global mapped climatologies of the period from 2002 to 2018 (NASA Goddard Space Flight Center), whereas pH values originate from the GLODAPv2 database [59, 60], with mean data displayed corresponding to the 0–30 m depths between 2000 and 2013. GLODAP, Global Ocean Data Analysis Project; MODIS, Moderate Resolution Imaging Spectroradiometer; NASA, National Aeronautics and Space Administration; PNG, Papua New Guinea; SST, sea surface temperature.

**Fig 2:**
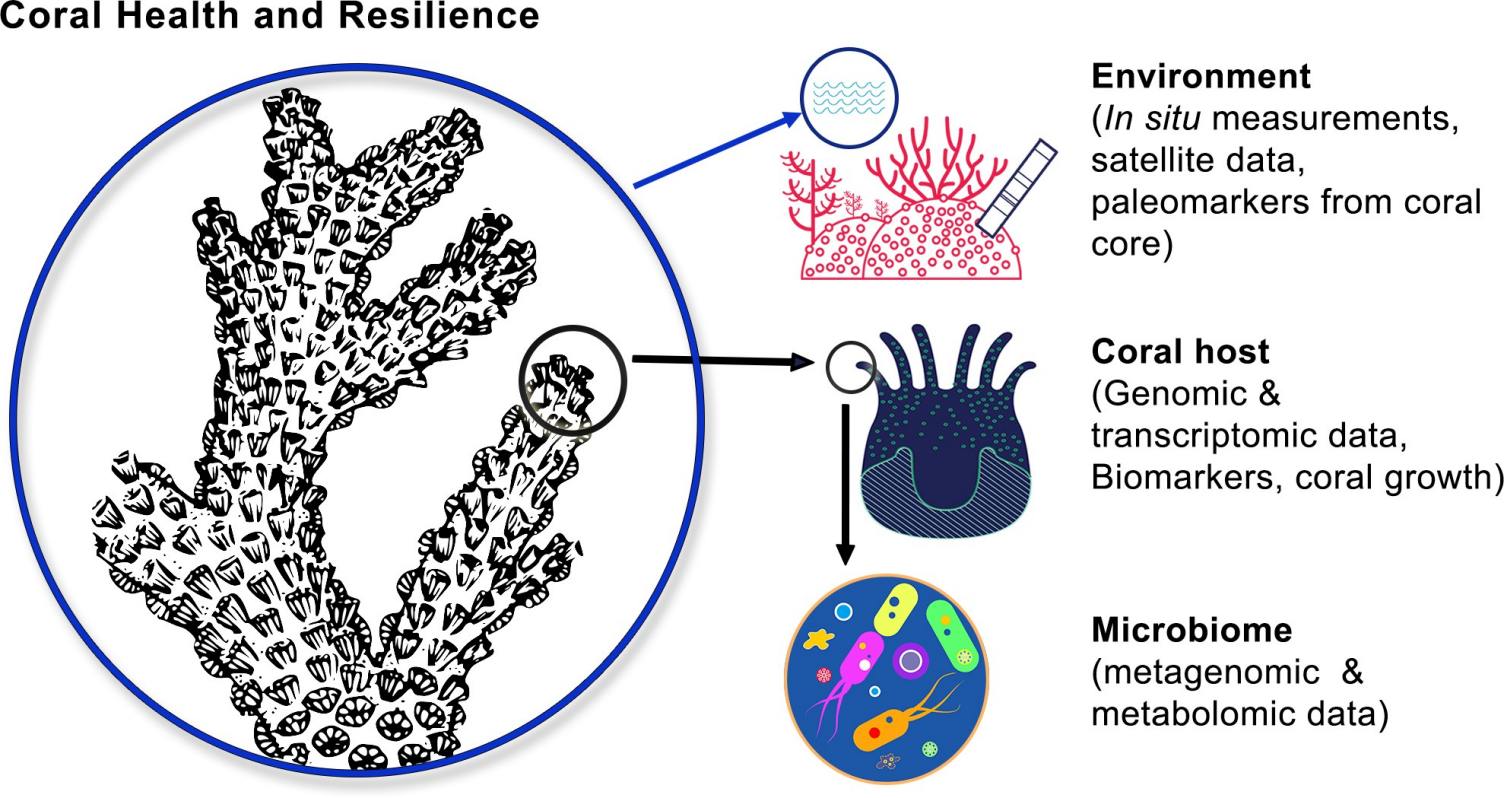
Schematic representation of the frame and major goals of *Tara* Pacific, which is investigating jointly the coral, microbiome, and environment.

The *Tara* Pacific expedition has built on these early tracks, applying the most recent technologies to map the “-omics” complexity of the coral holobiont within its ecosystem and across the Pacific Ocean. Through the exploration of marine biodiversity at scales spanning from organisms to genes to biomolecules, *Tara* Pacific is undertaking the first pan-ecosystemic study of coral reef diversity across an entire ocean basin (Fig 1). Given that a holistic approach integrating all components of the reef biota is unrealistic, the *Tara* Pacific expedition focuses its approach on widely distributed coral and fish holobionts and their contextual biological (plankton) and physicochemical environment, including modifications in the context of global changes (Fig 2).

The geographic and sequencing extent of *Tara* Pacific will bring an exceptionally comprehensive description of coral holobionts, together with the surrounding fish and plankton biota. Such unprecedented sequencing coverage and depth will place coral reefs among the first ecosystems with a comprehensive description of their hologenomic diversity (i.e., the totality of genomes making up the coral holobiont) across their natural environments. We will go beyond the mere description of species composition to investigate in detail the gene content and gene expression of these communities, as well as the interconnectedness with their hosts and habitats. Broad emerging patterns will then be accessible through large-scale data mining and network analyses.

## The coral holobiont

### The coral host

Scleractinian corals evolved over 450 million years ago [14] and belong to the phylum Cnidaria, animals located close to the root of the metazoans. Because of their place in the early Eumetazoa and as a sister group of bilaterians, the cnidarians allow interesting evolutionary analyses regarding the origins of animal complexity and the coevolution of early animals with microbes. Shinzato and colleagues [15] were the first to show that the coral genome is as complex as the one of vertebrates and that it has retained many ancestral genes lost in other lineages. Subsequent studies confirmed the deep genomic differences between coral taxa [16]. Moreover, the exceptional longevity of corals, reaching several hundred to thousands of years, makes them attractive emerging models for aging studies.

### The coral endosymbiont microalgae

Reef corals host symbiotic unicellular algae of the family Symbiodiniaceae inside their endodermal cells [17], raising intriguing questions on animal–plant interactions, photosymbiosis, coevolution, and speciation [18]. The divergence and genetic variation within and between populations of coral species is also investigated, allowing us to firmly ground the exact taxonomic status of each coral host [19].

### The coral microbiome

In addition to the Symbiodiniaceae, corals host diverse groups of microorganisms, bacteria, archaea, fungi, and protists, together forming the so-called coral holobiont [13]. The importance of the microbiome in the normal steady-state functioning of corals or during environmental stress is not well understood, but recent research suggests that microbiome structure aligns with and contributes to stress resilience [20, 21, 22] (in particular, bleaching [23, 24]), as seen in humans [25]. Recent work provides evidence that corals have coevolved with certain groups of microbes [26–28], but the exact nature of these host–microbe symbioses and their involvement in coral resilience remains unclear. Therefore, *Tara* Pacific will draw, using marker gene and metagenomic/metatranscriptomic approaches, a nearly exhaustive census of the microbial diversity associated with targeted coral taxa, allowing the understanding of the major drivers of the microbiome diversity and its role in coral health, resilience, and evolution.

### The coral virome

Viruses infect all known cellular organisms and are the most abundant biological entities on our planet [29]. Yet even the basic diversity of viruses associated with marine species and habitats is shockingly unknown. This is particularly true in tropical reef systems, where research on viral abundance, diversity, and dynamics is still in its infancy (for review, see [30]). *Tara* Pacific is using metagenomics and microscopy to interrogate the diversity of viruses across Pacific waters and within the foundation species of coral reefs.

## Research objectives of *Tara* Pacific

Our goal is to unveil the entire organismal diversity of eukaryotes, prokaryotes, and viruses associated with the targeted coral and fish holobionts and their surrounding waters and to assess variation across the explored ecological and geographical gradients. The main objectives of *Tara* Pacific are as follows:

- Draw a near-exhaustive census of the biodiversity composing and surrounding coral holobionts. This includes the coral hosts, their endosymbiotic microalgae (zooxanthellae of the family Symbiodiniaceae), and the associated microeukaryotes, bacteria, archaea, and viruses found either as endo- or exosymbionts, or in the plankton in surrounding water, using a metabarcoding approach.
- Determine the spatial patterns of the holobionts and the environmental diversity within coral reefs throughout the Pacific Ocean and derive large-scale biogeographic patterns to be compared with macrofauna patterns of diversity (i.e., the Pacific biodiversity gradient).
- Compare metatranscriptomes and metagenomes of coral reefs throughout the Pacific Ocean and elucidate the contribution of microbial diversity to local versus basin-scale adaptation in response to climate change. Importantly, the entire plankton community surrounding the corals and the islands has been sampled and integrated with physicochemical contextual data, providing critical context to advance our understanding of the environmental contribution to coral reef resilience.
- Investigate divergence and standing genetic variation within and between populations of the targeted coral species and their Symbiodiniaceae symbionts across the Pacific Ocean basin via marker gene barcoding as well as genome and transcriptome sequencing (population genomics using SNPs). Extensive metadata resources collected during *Tara* Pacific will allow the coral holobiont composition to be modeled, taking environmental and coral trait data into account (e.g., temperature, pH, bleaching, symbiont acquisition, reproduction cycle, etc.) to identify drivers of adaptation/selection or acclimatization, providing insights into coral stress resilience.
- Investigate the health status of corals by measuring growth parameters of the recent coral skeleton and stress biomarkers (antioxidant capacity, apoptosis, stress response pathways, transcriptomic signatures) as well as by determining the telomere status of each sample (telomeric DNA length and damage as potential proxies for stress). These data will be studied in relation to holobiont biodiversity, transcriptomic and metabolomics data, modern and/or historical environmental parameters, and stresses determined from a geochemical analysis of the coral skeleton.
- Generate a holistic coral metabolome for coral species as a foundation to the identification of the links between holobiont metabolism and prevailing environmental conditions.
- Identify how the environment (physical, chemical, biogeochemical, and biological) is influencing coral holobiont diversity, physiology, and evolution using an extensive compilation of environmental conditions.

## A unique, pan-ecosystemic sampling strategy

The Pacific Ocean covers approximately one-third of the earth's surface, with nearly 25,000 islands, most of which harbor coral reefs. *Tara* Pacific equipped the schooner *Tara* to explore 32 islands across the entire Pacific over a period of 2.5 years (Fig 1). The route of the *Tara* schooner was chosen to maximize the number of visited remote islands and atolls and to perform the widest possible comparative survey, from the equator to the temperate and more acidic regions, encompassing most of the environmental range where scleractinian coral species can live. It also covers a biodiversity gradient, from the low diversity present in the eastern Pacific reefs to the highly diverse western Pacific “warm pool” [14]. This sampling strategy also encompasses a wide variety of environments, from high temperature/low seasonality to low temperature/high seasonality, and also a full range of trophic status and physicochemical environmental parameters that affect coral reef ecosystems (sea surface temperature [SST], pH, nutrients, lights, pollutants, etc.).

**Fig 3:**
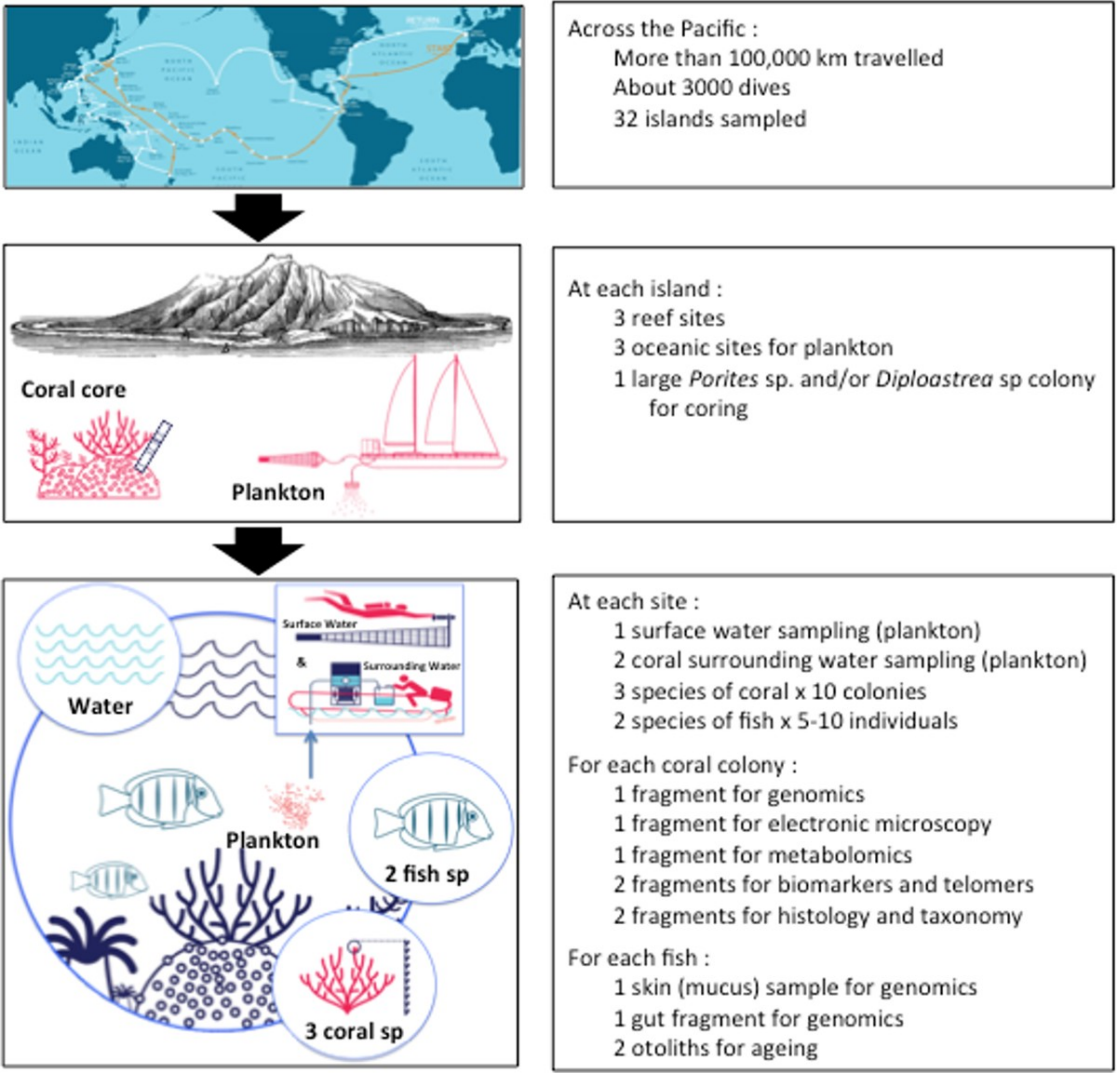
Schematic representation of the sampling design allowing the comparison of various components of coral reef ecosystems. Using the developed protocol, we collected 2,500 oceanic samples (32 islands × 3 oceanic sites × 5 size fractions × 2–5 protocols), 7,500 coral-surrounding and surface water samples (32 islands × 3 coral sites × 3 environments × 5 size fractions × 2–5 protocols), 40 coral core samples (32 islands × 1 *Porites* sp. and/or *Diploastrea sp* × 1–2 core samples), 20,160 coral fragments (32 islands × 3 coral sites × 3 species of corals × 10 colonies × 7 protocols), and 9,600 fish tissue samples (32 islands × 3 coral sites × 2 species of fish × 5–10 individuals × 5 protocols). Taken together, the dataset comprises a total of approximately 40,000 samples. *Bottom figure copyright to Agence DATCHA/taraexpeditions.org*.

Across its entire route, the *Tara* Pacific expedition targeted two species of scleractinian corals (Cnidaria, Anthozoa: *Pocillopora meandrina* and *Porites lobata*), one species of hydrocoral (Cnidaria, Hydrozoa: *Millepora platyphylla*), and two species of reef fish (*Acanthurus triostegus* and *Zanclus cornutus*) (Fig 3). The chosen taxa are among the few species that occur across most of the Pacific Ocean and are usually abundant on reefs. Around each of the 32 islands, three sites were sampled, collecting 10 colonies of each coral species and 5–10 individuals of each fish species, as well as water samples (see below) (Fig 3). Coral fragments and fish samples were preserved immediately on board, using specific buffers (e.g., DNA/RNA shield for genomics, glutaraldehyde for microscopy, etc.), and/or flash frozen for subsequent laboratory analyses (e.g., metabolomics, biomarkers, etc.).

In addition, seawater biogeochemistry and plankton microbiomes (viruses to zooplankton) were assessed in oceanic waters upstream and downstream of each island, as well as from water above each site (i.e., “surface water”) and surrounding two colonies of sampled *P*. *meandrina* corals at each site (i.e., “coral-surrounding water”). Underwater propeller-driven plankton nets (for surface water) and water pumping systems (for coral-surrounding water) were developed to collect plankton on reef sites, whereas the preparation of plankton samples into different size classes (<0.2, 0.2–3, 3–20, 20–2000 μm) and their preservation followed the *Tara* Oceans protocols [31] in order to maximize comparability and integration of data. Samples will be analyzed using high-throughput imaging and genomic techniques.

Finally, key physical, chemical, and biological parameters were measured continuously from surface water and from aerosols using specific instruments installed on board (e.g., thermosalinograph, mass- and spectrophotometers), as well as samples for imaging and genetic analysis of aerosol particles. Environmental context will also be obtained from satellite images and operational oceanographic products from the European Copernicus Marine Service and Mercator Ocean.

## *Tara* Pacific samples and data resources for the immediate and long-term future

*Tara* Pacific endorses the findable, accessible, interoperable, reusable (FAIR) principles for scientific data management [32] as well as ethical and responsible use of data. With a few exceptions, all physical samples of coral, plankton, fish, and sediments were preserved on board *Tara* and sent back to partner laboratories for subsequent analyses. Controlled vocabularies describing sampling devices and sample preparation protocols were used throughout the expedition to capture provenance metadata on customized log sheets, and samples were assigned unique identifiers to facilitate their traceability. Legal documents regulating the collection, export, and import of samples, as well as links to the Convention on Biological Diversity’s (CBD) access and benefit-sharing clearing house (https://absch.cbd.int/) are in the process of being provided for each sample as a first step toward ethical and responsible use of the *Tara* Pacific data. The detailed registry of all samples, including their provenance and environmental and legal context, is curated manually using simple semantics that enable machine- and human-readable data discovery services. Sequencing data will be deposited at the free, open-access European Nucleotides Archive (https://www.ebi.ac.uk/ena); environmental data are deposited at the free, open-access PANGAEA database (https://www.pangaea.de/); and both archives will be interlinked *via* the sample registry available online at BioSamples (https://www.ebi.ac.uk/biosamples/). Metabolomic data (mass spectrometry [MS] and nuclear magnetic resonance [NMR]) and their annotations will also be accessible through the Metabolights portal (https://www.ebi.ac.uk/metabolights/). The submission of data in a relational, open-access, updated, and cured database is critical and necessary for our aim to establish a reference of the biological state of coral reefs in the Anthropocene for the broader research community.

## Metagenomics and metatranscriptomics to explore the diversity and physiology of coral reef holobionts

Building upon expertise gained in the previous *Tara* Oceans project [33–36], we are applying Illumina HiSeq technology to sequence a series of barcodes designed recently to explore the diversity of bacteria and archaea (V4–V5 region of the nuclear 16S rRNA gene [37]), eukaryotes (V9 region of nuclear 18S rRNA gene [33]), Symbiodiniaceae (ITS2 region of the nuclear ribosomal DNA [38–40]), and metazoan species (mitochondrial COX1 gene [41]), as well as metagenome assembly–defined viral populations [42, 43]. Barcode-specific PCRs are performed on DNA from coral tissues, coral-surrounding water, fish tissues, and surface water above the reef in order to assess interkingdom diversity associated with the holobiont at different degrees of proximity from the coral animal host, from endosymbiotic to drifting in coral-surrounding and surface waters. Biogeographic gradients will also be investigated between the studied island systems. The sequence reads are assembled into operational taxonomic units (OTUs) for bacteria, archaea, and eukaryotes and into ITS2-type profiles (using the SymPortal framework at symportal.org [40]) for Symbiodiniaceae and taxonomically annotated by comparison to reference databases. Classical numerical ecology methods are applied to employ OTU richness and abundance data to (1) assess and compare the total diversity of bacteria, archaea, and eukaryotes associated with various compartments of the different coral holobionts; (2) compare biodiversity patterns across coral colonies, hosts, reefs, islands (geographic and Lagrangian oceanic circulation distances), and all contextual environmental parameters measured; (3) integrate the *Tara* Pacific data into the *Tara* Oceans global plankton dataset. In addition, co-occurrence graph techniques inspired from systems biology [44, 9] are used to reconstruct OTU interaction (sub)networks, and we will aim to disentangle the intrinsic (symbiotic auto-organization) from the extrinsic (environmental, historical contingencies) forces on holobiont biodiversity. Overall, this study is the first attempt to characterize coral reef and holobiont diversity across comprehensive taxonomic, ecological, and geographical scales over an entire oceanic basin. Comparison with *Tara* Oceans plankton data will also reveal which part of the coral holobiont biodiversity is found in the open ocean and thus bring fundamental information about reef connectivity, as recently shown for Symbiodiniaceae, which are found all over the plankton [34].

The sequencing of the holotranscriptomes of coral holobionts sampled along the transect will provide important genetic information to uncover ecological and evolutionary questions at both intraspecific and community levels. Two protocols are successively applied on total RNA from three specimens per species and per site. In a first step, a polyA+ enrichment protocol is applied to provide eukaryotic mRNA in order to study the coral and Symbiodiniaceae dual transcriptome, and in a second step, rRNA is removed from the remaining polyA− fraction to sequence microbial mRNA. We are applying different metatranscriptomics protocols to capture the gene expression of the Cnidaria, Symbiodiniaceae, bacteria and archaea, other microbes, and even viruses from the same initial tissue samples [45, 46]. In addition, we are using similar analysis procedures after filtration of coral-surrounding water samples. This may potentially reveal how microbes and viruses interact with the coral host and how the coral host adapts or acclimatizes to their presence. On top of providing deep insight on gene expression networks at the holobiont level, mRNA samples will also be used to generate transcriptome-wide SNPs for the coral host, allowing in-depth characterization of the patterns of diversity, connectivity, demographic history, and local adaptation of sampled coral species. Hence, the resulting patterns will provide a solid foundation on which to base the study of partner relationships within the coral holobiont, and possibly even allow for the identification of the selective forces acting on key genes for these symbiotic associations, plus an account on standing genetic diversity within and between reefs to highlight regions of high genetic endemicity and diversity important for conservation efforts.

## A metabolomic approach

We will also assess the metabolome (i.e., the metabolic diversity) of the three targeted coral species (two stony and one fire coral, see above). Small molecules (i.e., specialized metabolites) are the end products of unique metabolic pathways. Most of these presumably possess an ecological role, and as such, they represent key phenotypic traits for the specimens. The broad geographical cover of the collected coral species will enable us to provide some insight into the relative contribution of environmental factors and the genetic information expressed in the metabolome by comparison with genomic data. Recent advances in analytical techniques and bioinformatics led to the development of global metabolomics approaches capable of providing an overview of the thousands of metabolites present in a minute amount of sample [47, 48]. A nontargeted metabolomics approach is applied to the three species of corals collected across the Pacific Ocean using both MS and NMR. The subsequent identification of the chemomarkers unveiled by these comparative approaches are performed using not only databases of experimental spectra but also comparison with in silico databases of metabolites. The results will finally contribute to the assessment of the reef health status when combined with other “-omics” data.

## Identifying the interaction among environmental stressors, the holobiont, and coral resilience

A key question to be addressed by *Tara* Pacific is how reef corals change geographically and how corals locally adapt or acclimate to environmental changes and increasing stress. This question has been rarely studied at the scale of a complete ocean and across multiple populations under consideration of phenotypic traits [49]. The physiological status of corals will be assessed by measuring the parameters of coral growth such as density, linear extension, and calcification rates of *P*. *lobata* (with added *Diploastrea heliopora* in few places) colonies by analyzing coral cores recovering the last 50–150 years by sclerochronology. The annual density bands and growth parameters of the massive coral sampled along the transect will be determined using X-ray radiography and 3D-computed tomography of growth rings on sliced coral cores [50–52], allowing the calculation of recent reef growth over the last decades and century. In parallel, ambient sea temperature and pH changes over the same period of time will be documented by analyzing, at an annual or monthly timescale, the most advanced geochemical tracers along the cores, such as boron isotopes or trace elements, trapped along the cores during the coral life [53–56]. Recent stress events will be identified by a series of conventional markers of physiological stress or damage (see above) [57]. In addition, telomere length measurements of coral and microalgal symbiont genomes will be conducted. Notably, although measurements of telomere length as a method of assessing stress accumulation is currently being employed in numerous human cohort studies, the importance of telomere length for stress resilience and biodiversity in ecological studies is in its infancy and remains to be determined [58]. Thus, it will be highly informative to compare telomere length variations with coral growth measurements assessed by sclerochronology.

More broadly, the integration of environmental and physicochemical with biological data will allow us to pinpoint the specific adaptations that enable corals to live and survive across environmental gradients. Comparison of current to historical data throughout the Pacific will then show the biological cost of this adaptation and the associated time span. This in turn will make for a much better understanding of the capacity of coral holobionts to adapt to adverse environmental conditions and the required time scales.

In summary, *Tara* Pacific is poised to build the most comprehensive morphomolecular inventory of the phenotypic and genotypic biodiversity of coral reef ecosystems, including a wide spectrum of life from viruses to bacteria and from unicellular eukaryotes to metazoans and covering nested spatial scales from coral holobiont colonies, their surrounding water (i.e., coral-surrounding water), reef surface water, and neighboring oceanic surface waters (upstream and downstream of the sampled islands). The project focuses on selected key species that are ubiquitous in coral reef ecosystems throughout the Pacific and can be traced from an environmental, physicochemical, biological perspective down to the gene. This ambitious project has the potential to reveal substantial uncharted biodiversity; to shed light on the complex links between genomes, transcriptomes, metabolomes, organisms, and ecosystem functions in coral reef systems; and to provide a basis for the biodiversity and biological state of modern coral reefs for the research community at large. In addition, this project will contribute significantly to other fields of research such as stress and aging biology. We expect that the unprecedented scale of this project will help to decipher the complex interactions that together ensure a healthy state of coral reefs, a quest for which advances are urgently needed.

## Abbreviations

CBD: Convention on Biological Diversity
FAIR: findable, accessible, interoperable, reusable
GLODAP: Global Ocean Data Analysis Project
MODIS: Moderate Resolution Imaging Spectroradiometer
MS: mass spectrometry
NASA: National Aeronautics and Space Administration
NMR: nuclear magnetic resonance
OTU: operational taxonomic unit
SST: sea surface temperature
